# Conquering the beyond Rule of Five Space with an Optimized High-Throughput Caco-2 Assay to Close Gaps in Absorption Prediction

**DOI:** 10.3390/pharmaceutics16070846

**Published:** 2024-06-22

**Authors:** Patricia Muschong, Khader Awwad, Edward Price, Mario Mezler, Manuel Weinheimer

**Affiliations:** 1 Quantitative, Translational and ADME Sciences, AbbVie Deutschland GmbH & Co. KG, Knollstrasse, 67061 Ludwigshafen, Germany; patricia.muschong@abbvie.com (P.M.); khader.awwad@abbvie.com (K.A.); mario.mezler@abbvie.com (M.M.); 2 Research and Development, AbbVie Inc., 1 North Waukegan Road, North Chicago, IL 60064, USA; edward.price@abbvie.com

**Keywords:** Caco-2, beyond rule of five (bRo5), proteolysis targeting chimera (PROTAC), absorption, permeability, in vitro assay, oral bioavailability, f_a_, fafg

## Abstract

Current drug development tends towards complex chemical molecules, referred to as “beyond rule of five” (bRo5) compounds, which often exhibit challenging physicochemical properties. Measuring Caco-2 permeability of those compounds is difficult due to technical limitations, including poor recovery and detection sensitivity. We implemented a novel assay, with optimized incubation and analytics, to measure permeability close to equilibrium. In this setup an appropriate characterization of permeability for bRo5 compounds is achievable. This equilibrated Caco-2 assay was verified with respect to data validity, compound recovery, and in vitro to in vivo correlation for human absorption. Compared to a standard assay, it demonstrated comparable performance in predicting the human fraction absorbed (f_a_) for reference compounds. The equilibrated assay also successfully characterized the permeability of more than 90% of the compounds analyzed, the majority of which were bRo5 (68%). These compounds could not be measured using the standard assay. Permeability and efflux ratio (ER) were highly predictive for in vivo absorption for a large set of internal bRo5 compounds. Reference cut-offs enabled the correct classification of high, moderate, and low absorption. This optimized equilibrated Caco-2 assay closes the gap for a high-throughput cellular permeability method in the bRo5 chemical space.

## 1. Introduction

In the small molecule chemical space within Lipinski’s rules of five (Ro5) [1], compounds exhibit a high range of solubility/permeability, which can be correlated reasonably well to the fraction absorbed (f_a_). For cellular permeability (P_app_), this correlation represents a sigmoidal curve, where highly absorbed compounds mostly demonstrate high permeability [2]. However, for compounds with a P_app_ < 10 × 10^−6^ cm/s, the accuracy to predict f_a_ decreases, and for permeabilities < 1 × 10^−6^ cm/s, traditional cellular P_app_ becomes merely a qualitative marker (low permeability = likely poorly absorbed). Unfortunately, the majority of more complex small molecule drugs beyond the rule of five (bRo5), such as proteolysis-targeting chimeras (PROTACs), fall within this range [3]. Besides a lack in predictability, traditional cellular permeability assays are often not able to generate a permeability value, because of low detection sensitivity due to the very low permeability, as well as nonspecific binding, of the compound to the incubation setup.

Novel in silico-based and partitioning-based approaches, such as AB-MPS-score [4], Lipophilic Permeability Efficiency (LPE) [5], ChameLogD, Experimental Polar Surface Area (EPSA) [6], Chamelogk [7], and the EPSA-to-TPSA Ratio (ETR) [8], provide high-throughput support for bRo5 projects, especially at early stages [9].

These strategies either address multiple physicochemical parameters in parallel or consider the conformational change of a compound to reduce polarity during membrane passage, often coined “chameleonicity”. Such polarity-reducing effects can lead to a better permeation and absorption of bulky molecules [8].

Although these models provide a physicochemical representation of permeability for early compound ranking and absorption prediction, they overlook the complexities of biological behavior caused by para- and transcellular transport, compartmental binding, or active transport, and may need a cellular system to better predict permeability and absorption. As the standard cellular transwell^®^ approach is not suitable for compounds with very low permeability and unfavorable physicochemical properties, previous studies aimed to modify the setup to enable better compound recovery and sensitivity. These approaches included the use of bovine serum albumin (BSA) in the transport medium [10] or the prolongation of the incubation time within the assay. To mirror the physiological lag time of slowly permeating compounds, pre-incubation steps can assure the measurement of permeability at steady state. These improvements have been shown to better characterize the transport of bRo5 compounds [11].

The goal of the present study was to identify the most suitable conditions for a simple cell-based assay that provides superior performance with respect to the characterization of permeability, the modeling of gut absorption, and to improve overall data quality and reproducibility.

Therefore, we investigated the relevance of a pre-incubation step, optimized LC-MS/MS analytics, and the addition of BSA for the measurement of permeability of complex chemical entities. To verify the suitability of the setup, we analyzed 61 known reference compounds with available human f_a_. Lastly, we measured the permeability and efflux of 741 internal compounds, many of which violated two or more Lipinski rules (bRo5) and compared the results to rodent absorption.

## 2. Materials and Methods

### 2.1. Materials

Unless stated otherwise, all compounds including inhibitors were obtained internally from AbbVie chemical substance management system (Ludwigshafen, Germany). Assay-ready Caco-2 cells were purchased from acCELLerate (Hamburg, Germany). Buffers and cell culture reagents, e.g., Hank’s Balanced Salt Solution (HBSS), Dulbecco’s Phosphate Buffered Saline (DPBS), trypsin/EDTA, Dulbecco’s Modified Eagle Medium (DMEM), sodium pyruvate, L-glutamine, non-essential amino acids (NEA), fetal bovine serum (FBS), and bovine serum albumin (BSA) were all purchased from Thermo Fisher Scientific (St. Leon-Rot, Germany). All plasticware was internally available in AbbVie.

### 2.2. Cell Culture and Plate Preparation

Frozen assay-ready Caco-2 cells [12] in cryovials were taken from liquid nitrogen storage and thawed in a water bath at 37 °C for 2 min. The cells were transferred into a 50 mL Falcon tube and 8 mL of prewarmed cell culture medium (DMEM supplemented with 10% FBS, 1% NEA, 1% L-glutamine, and 1% sodium pyruvate) was added. The cell suspension was centrifugated at 80× *g* for 4 min and the supernatant cautiously aspirated. The cell pellet was resuspended in 10 mL of fresh cell culture medium and mixed gently. After equilibration for 30 min at room temperature, the cells were seeded into transwell plates.

The cells were seeded into 0.4 µm Millicell^®^ 96-well transwell plates (Merck Millipore, Darmstadt, Germany), comprising the insert plate and the feeder plate. The insert plate was seeded with 100 µL/well cell suspension containing 40,000 cells. The feeder tray was filled with 28 mL of medium. Unless stated otherwise, Caco-2 cell monolayers were grown for 7–8 days at 37 °C with 5% CO_2_. Medium was changed on the basolateral side 4–5 days after seeding and apically one day before the experiments to prevent cell starvation. The plate inserts were then transferred into 96-well receiver plates (Merck Millipore, Darmstadt, Germany) filled with 200 µL/well fresh culture medium.

### 2.3. Bidirectional Transport Studies

If not described differently, compounds were dissolved in dimethyl sulfoxide (DMSO) to 10 mM stock solutions and further diluted in HBSS at pH 7.4 containing the monolayer-integrity marker lucifer yellow (80 µM final conc.) to the required concentrations. Depending on the assay modifications, 1% (*w*/*v*) BSA was added to the HBSS buffer. Final DMSO concentrations were max 0.2% (*v*/*v*).

Before the assay, culture medium was removed, and the cells were rinsed with HBSS (pH 7.4) once. Compound solutions (1 µM or 3 µM) were added to donor compartments, i.e., apical side of A-to-B transport direction and basolateral side of B-to-A direction. The receiver compartments were filled with corresponding receiver buffer (HBSS pH 7.4 with or without 1% BSA). Compound permeability was evaluated after 60 min incubation at 37 °C, when samples were collected from both apical and basolateral sides of both transwell plates, and then mixed with a quench solution (30% acetonitrile in water or 100% ethanol, containing 25 nM carbutamide as internal LC-MS/MS process control) before measurement using LC-MS/MS. When conducting a pre-incubation step, compound solutions were added to donor compartments and the receiver compartments were filled with corresponding receiver buffer (HBSS pH 7.4 with or without 1% BSA). Unless stated otherwise, the pre-incubation solution was removed after 60–90 min. The cells were then rinsed with HBSS with 1% BSA, and new compound solution (donor compartments) and receiver buffer (receiver compartments) were added for the main incubation (60 min) with subsequent sampling for permeability assessment. To determine compound apparent permeability (P_app_), the following equation was used:

(1) $$P_{app}=\frac{∆Q}{∆t\cdot A\cdot \left(\frac{c_{1}+c_{0}}{2}\right)}$$

where ΔQ is the amount of compound permeated through the monolayer as determined by the response (=peak area) of compound in the receiver well at the end of the experiment, Δt is the incubation time in s, A is the filter surface area (0.11 cm^2^), C_1_ is the response in the donor well at the end of the experiment, and C_0_ is the initial nominal compound concentration. Permeation velocity is expressed as P_app_ with the unit of 10^−6^ cm/s.

Efflux ratio (ER) was calculated as follows:

(2) $$ER=\frac{P_{app,BA}}{P_{app,AB}}$$

where P_app,AB_ and P_app_,_BA_ refer to the mean permeability of two replicates in the direction of apical to basolateral (A-to-B) or basolateral to apical (B-to-A), respectively.

Recovery (%) was determined using the following equation:

(3) $$Recovery(\%)=\frac{C_{Acceptor}+C_{Donor}}{C_{0}}\cdot 100$$

C_Acceptor_ and C_Donor_ are the responses of compound determined in the acceptor and donor well at the end of the incubation time, respectively. C_0_ is the initial nominal compound concentration.

All studies were performed at least in technical duplicates. Mean values were used for final data analysis.

### 2.4. Analytical Method

All measurements were performed using an Acquity Iclass UPLC (Waters, Milford, MA, USA) system coupled with a tandem mass spectrometer Sciex 6500 (Sciex, Framingham, MA, USA) operating dependent on the tested compounds in positive or negative mode. Chromatographic separation of a 1–7.5 µL injected sample was achieved with a BEH C18 column (2.1 mm × 30 mm, 1.7 μm) kept at 60 °C. The total run time was a linear 1.1 min gradient starting for most tested compounds with 95% water (mobile phase A) and 5% acetonitrile (mobile phase B), both acidified with 0.1% formic acid and peaking with 95% mobile phase B. Optimized mass transitions were used to detect incubated substances. Acquisition and analysis of the data were performed using Analyst 1.7.2 and Discovery Quant 3.0.1 (Sciex). Variability within and across assays was assessed using carbutamide as an analytical process control substance. Results were reported as peak area (response). For compound optimization, a 40 nM test solution was injected and optimized in terms of chromatographic separation, ionization, and fragmentation. A signal-to-noise ratio of at least 3 compared to blank injection was considered as appropriate. The analytical threshold for detection was considered sufficient when a minimum peak area in the donor compartment to enable a P_app_ of 10^−6^ cm/s (or 10^−7^ cm/s) was theoretically achievable. This translated to a peak area threshold of at least 300,000 or 3,000,000, respectively, for the 40 nM test solution, assuming comparable matrix conditions and extraction efficiencies in the donor and acceptor.

During compound optimization, a series of alternative chromatographic separations were tested and used if binding (fronting) or retention (tailing) was reduced or signal-to-noise for a 40 nM test solution increased, compared to the standard method described above. The adaptions contained but were not restricted to different column chemistry (Acquity Xbridge C8 (Waters, 2.1 mm × 30 mm, 3.5 µm)) or the replacement of mobile phase A with 25 mM ammonium bicarbonate supplemented with 25 mM ammonium hydroxide. For tacrolimus, chromatography was a linear 1.1 min gradient starting with 50% 10 mM ammonium formate (mobile phase A) and 50% acetonitrile (mobile phase B), both acidified with 0.1% formic acid and peaking with 98% mobile phase B.

### 2.5. Detailed Workflow of the Final Assay Setup

Three Caco-2-based transport study designs serve as the basis for comparing in vitro systems in this study (Table 1). Our goal was to develop an assay with optimized throughput and robust turnaround time. As a first step to achieve this goal, we utilized assay-ready cells for a shortened cultivation period of 7 days. The comparison with the commonly used 21-day cultivation time showed highly comparable outcomes in terms of permeability and efflux (Figure S2). By modifications and comparisons with compounds of known absorption, the study parameters described in 2.3 have been identified as a superior design (Figure S1).

The standard method is designed as a high-throughput assay with a short incubation time and without the use of BSA. It can accommodate up to 3 compounds cassetted at an individual concentration of 1 µM, allowing for a capacity of 132 compounds/assay along with four reference standards.

The BSA-modified standard method aims to characterize compounds that are not accessible with the standard method. In addition to adding 1% BSA to the assay buffer to solubilize compounds and to prevent non-specific binding, higher concentrations of 3 µM are used in a non-cassetted approach. This assay provides a lower throughput (44 compounds/assay).

**Figure 1:**
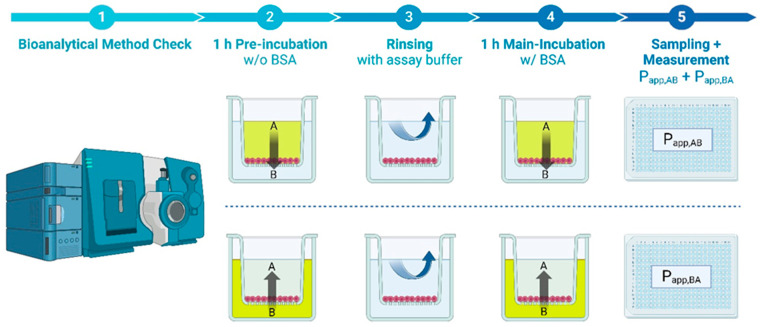
Detailed assay workflow of the equilibrated Caco-2 permeability assay in its final setup. The assay workflow starts with an initial bioanalytical method check (LC-MS/MS) of the test articles on a Waters Acquity coupled to a Sciex 6500 (1). Caco-2 cells in transwell plates are pre-incubated apically or basolaterally with compound at 3 µM in HBSS for 60 min (2). After rinsing of the cells with assay buffer (3), the 60 min main incubation with 3 µM compound in HBSS + 1% BSA follows (4). After the main incubation, samples are taken and measured via LC-MS/MS (5). Image created with BioRender.com.

Lastly, we introduced an equilibrated method that includes a pre-incubation step to saturate the system and achieve better compound recovery. This method mimics steady-state permeation, which is particularly important for bRo5 compounds with high molar mass, such as PROTACs. To further optimize this assay, the analytical method was improved to enhance sensitivity, as previously described (2.4). Like the BSA-modified standard method, this approach covers 44 compounds/assay. The workflow of the equilibrated method is depicted in Figure 1.

### 2.6. Evaluation of Pre-Incubation Modification for the Equilibrated Method

**Figure 2:**
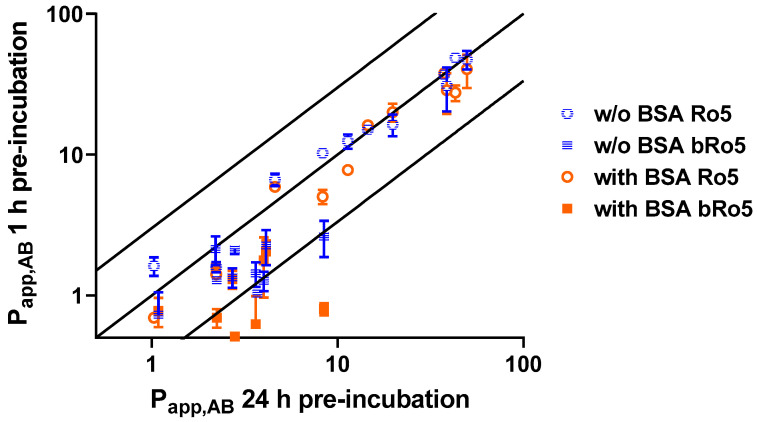
Investigations of different pre-incubation regimes. Pre-incubation for 1 h with and without BSA was compared to 24 h pre-incubation as described by Cui et al. [11] for a set of reference and internal compounds covering Ro5 and bRo5 (Table 2, Methods 2.6). The main incubation was performed with BSA for 1 h in all conditions. Lines indicate line of unity ±3 fold.

We investigated the pre-incubation process, focusing on the use of BSA as a supplement in the buffer. A set of 21 representative compounds (Ro5 and bRo5; Table 2) was tested in either setup and permeabilities were compared to a full 24 h pre-incubation setup as a control, accounting for total equilibration of the system, as described by Cui et al. [11]. Based on these results, the final setting of the equilibrated method was established without the use of BSA in the pre-incubation but with BSA in the main incubation (Figure 2).

### 2.7. In Vivo Absorption

To compare the in vitro results with in vivo absorption, we utilized either known literature human fraction absorbed (f_a_) or approximated internal rodent fafg, assuming the fraction escaping gut metabolism (f_g_) to be close to 1. The reference compounds included in this study are listed in Table 3.

The fafg values were obtained from internal rodent (mouse and rat) in vivo intravenous (IV) and oral (PO) pharmacokinetic studies. Specifically, the rodent IV blood or plasma clearance (Cl_B_ or Cl_P_) was used to calculate the hepatic bioavailability (f_h_) using the extraction ratio, assuming a blood to plasma ratio of 1:

(4) $$f_{h}=1-\frac{{Cl}_{B}\;or\;{Cl}_{P}}{Q_{h}}$$

Hepatic blood flow (Q_h_) has been estimated, based on internal and external sources, to be 5.2 L/h/kg for mice and 3.8 L/h/kg for rats [13,14,15,16]. The area under the concentration vs. time curves (0 to t) were dose-normalized and used to calculate the total oral bioavailability, F_PO_.

(5) $$F_{PO}=\frac{{AUC}_{PO}\times D_{IV}}{{AUC}_{iv}\times D_{PO}}$$

where AUC is the area under the blood- or plasma-level curve and D the respective IV and PO doses.

Approximated intestinal absorption expressed as fafg was then calculated as follows:

(6) $$fafg=\frac{F_{Po}}{f_{h}}$$

**Table 3:** Reference compounds and their observed human f_a_ values. Human f_a_ values taken from [17]. Values were rounded to two significant figures.

Reference Compound	Observed Human f_a_
Amiloride	0.53
Amprenavir	0.76
Antipyrine	0.98
Azithromycin	0.43
Biperiden	1.0
Boceprevir	0.92
Bosentan	0.70
Bupropion	0.88
Caffeine	1.0
Cetirizine	0.73
Clozapine	0.99
Corticosterone	1.0
Diclofenac	0.97
Dipyridamole	0.56
Doxepin	0.76
Flecainide	0.82
Furosemide	0.61
Gliquidone	0.95
Hydroxychloroquine	0.90
Imatinib	0.90
Indinavir	0.63
Ketoconazole	0.76
Lamotrigine	0.98
Lenalidomide	0.90
Loperamide	0.52
Loratadine	0.90
Methotrexate	0.64
Metoclopramide	0.89
Mexiletine	0.99
Moclobemide	0.85
Naratriptan	0.70
Nicotine	1.0
Nimodipine	1.0
Nortriptyline	1.0
Ondansetron	1.0
Oseltamivir	0.75
Phenazopyridine	0.90
Phenytoin	0.93
Physostigmine	0.050
Pirenzepine	0.26
Propranolol	0.97
Ranitidine	0.56
Rifabutin	0.53
Riluzole	0.90
Risperidone	0.85
Rizatriptan	0.90
Rosiglitazone	1.0
Rosuvastatin	0.35
Roxithromycin	0.86
Selegiline	1.0
Sulfasalazine	0.33
Sumatriptan	0.58
Telmisartan	0.82
Trazodone	0.98
Trihexyphenidyl	1.0
Vardenafil	0.90
Venlafaxine	0.96
Verapamil	0.92
Vismodegib	0.69
Ziprasidone	0.63
Zolmitriptan	0.92

### 2.8. Statistical Analysis

Significance was tested based on Welch’s *t*-test assuming unequal variances, using GraphPad Prism Software (Version 9.5.0). Significance was determined based on *p* < 0.05 (*), *p* < 0.01 (**), *p* < 0.005 (***), and *p* < 0.001 (****).

## 3. Results

### 3.1. Comparison of Pre-Incubation Parameters

An important assay improvement is the pre-incubation step that addresses the equilibration of a compound, which is required to measure steady-state permeability. Cui et al. suggested a 24 h pre-incubation to reach full equilibrium [11]. However, such a long pre-incubation time is also considered challenging, especially due to potential cytotoxic effects of the compounds on the cells, or the potential degradation of the compound in the aqueous solutions or within the cells of the assay. Therefore, we investigated options to reach equilibrium permeability at shorter incubation times. To test this, we applied a pre-incubation step for 1 h only. An evaluation with or without BSA allowed us to also investigate the effect of protein on the recovery and permeability of the compound in the assay.

Figure 2 shows the P_app,AB_ of the reference compounds from Table 2 obtained with 1 h pre-incubation with and without BSA in comparison to 24 h pre-incubation (the main incubation was always conducted with BSA). For the Ro5 compounds, all results were within three-fold of unity as a benchmark of variability between assays. However, at 1 h pre-incubation with BSA, a decreased permeability was more often observed for bRo5 compounds compared to 24 h or 1 h without BSA. Altogether, differences between the pre-incubation regimes were more frequently noticed in the low-permeability space. Nevertheless, using an abbreviated 1 h pre-incubation without BSA compared well with the 24 h pre-incubation period. These data also align with the results demonstrated by Cui et al.

### 3.2. Assay Validity

**Figure 3:**
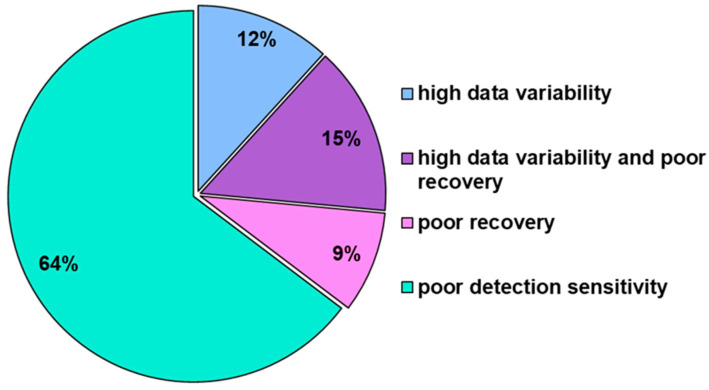
Reasons for invalid (NV) or qualified results in a compound set of 52 entities with broad physicochemical properties in the standard method. Poor detection sensitivity is indicated when the analyte concentration in the receiver is below the limit of detection (LOD) of the analytical method, resulting in the expression of the result as qualified P_app_, employing the LOD as a basis value for calculation. Poor recovery is indicated when the mass balance is less than 65% in a single direction, or less than 40% in bidirectional studies, where the recoveries are similar in both directions (with a recovery difference of less than 25%). High data variability is indicated when the difference between duplicates exceeds 50%, which is often observed in combination with recovery issues.

The biggest challenge in measuring cellular permeabilities of bRo5 compounds is to obtain accurate results. Due to their unfavorable physicochemical properties, bRo5 molecules typically exhibit lower permeabilities, resulting in lower concentrations in the acceptor compartment. In addition, non-specific binding to the assay plates and low solubility are big challenges, all leading to very low compound concentrations, particularly in the receiver well of a transwell setup. Low concentrations in the receiver well require a higher sensitivity of the analytical method. Furthermore, lower concentrations are even more susceptible to non-specific binding, which can lead to a concomitant reduction in compound recovery. The reasons for a de-validation of the results in the standard method (see description in 2.5) or the necessity to express the results with qualifiers are provided in Figure 3.

**Figure 4:**
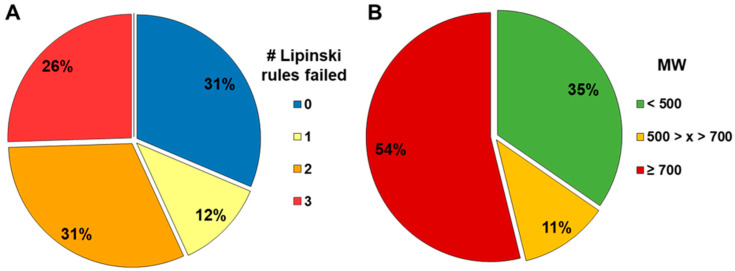
Distribution of compound types evaluated in all three methods based on the number of zero-to-three failed Lipinski rules (**A**) and the molecular weight (**B**).

Using our acceptance criteria (Figure 3), we compared a subset of 52 compounds from various compound classes regarding the ability to obtain either valid or qualified (lower than LOD) results. The compounds were tested in the standard method, the BSA-modified standard method, and in the equilibrated method (detailed assay description in Methods 2.5). A total of 57% of the compounds violated two or more of the Lipinski rules. Additionally, 65% had a molecular weight greater than 500 g/mol, and 54% had a molecular weight exceeding 700 g/mol (Figure 4).

**Figure 5:**
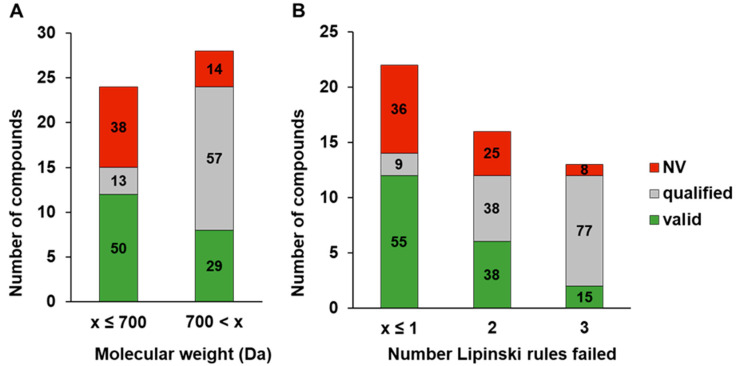
Qualifier distribution for the shared compound set in the Caco-2 standard method based on the molecular weight (**A**) and number of failed Lipinski rules (**B**). The labels represent the respective percentages of each bar.

It was demonstrated that both the molecular weight and the number of violated Lipinski rules were relevant for the proportion of valid and qualified results in the Caco-2 standard method (Figure 5). Compounds with a higher molecular weight and more Lipinski violations had a lower percentage (24% (Figure 5A) and 11 to 17% (Figure 5B)) of invalid results, but the percentage of qualified results increased (44% and 29 to 68%). This could be attributed to insufficient permeation of such compounds under the given conditions, as well as a very low concentration below the LOD in the acceptor compartment. Therefore, the compound-specific LOD determines the qualified value (Figure 3). In total, this leads to a decline in valid results from over 50% to 29% for compounds with molecular weights greater than 700, or to 38% and 15% if two or three Lipinski rules failed, respectively.

**Figure 6:**
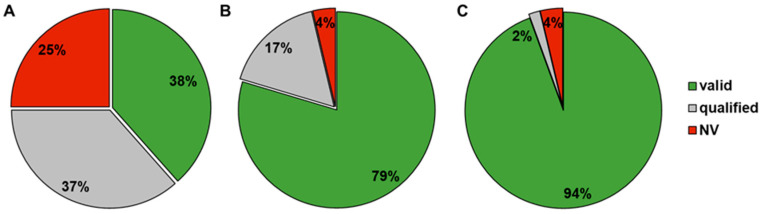
Distribution of valid, qualified, and invalid (NV) results based on a shared compound set for the standard method (**A**), the BSA-modified standard method (**B**), and the equilibrated method (**C**).

Overall, for this compound set, only 38% could be fully characterized with the standard method. For most compounds, the permeability was qualified (37%) or not valid (NV; 25%) (Figure 6).

**Figure 7:**
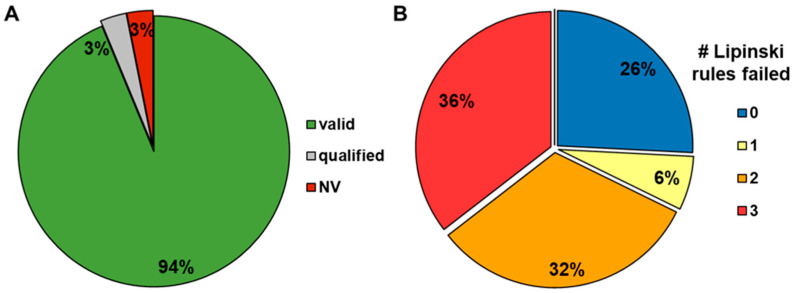
Distribution of valid, qualified, and invalid (NV) results obtained with the equilibrated method for those compounds with qualified and NV results with the standard method (**A**), as well as the respective numbers (0 to 3) of Lipinski violations (**B**) of the same compounds.

The addition of BSA to the assay buffer (BSA-modified standard method) in a single incubation of 1 h improved the rate of valid results to 79%, and with the pre-incubated setup (equilibrated method without BSA in the first and with BSA in the second incubation), a data validity of 94% was achieved (Figure 6). Furthermore, when considering only compounds that had a qualified or invalid result in the standard method (68% of them bRo5), the pre-incubated setup still covered 94% of that fraction (Figure 7).

### 3.3. Recovery

Compound recovery is considered an important quality criterion of an assay. A higher percentage of recovery (>80%) may be required to uphold the validity of the generated permeability data. This high level of recovery is also required by the regulators, e.g., in biowaiver studies [18], as the compound loss could account for non-specific binding to the assay setup, compound precipitation, or degradation of the molecule.

**Figure 8:**
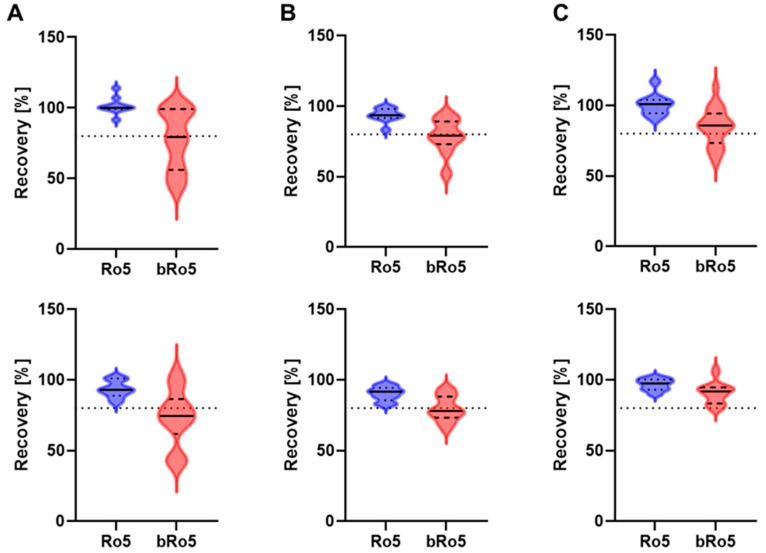
Violin plot analyses of compound recovery values generated with the standard method (**A**), the BSA-modified standard method (**B**), and the equilibrated method (**C**). Upper and lower rows display recovery values of A-to-B transport and B-to-A transport, respectively. The compound set comprised 27 compounds with 67% being bRo5. Recovery is marked in blue and red for Ro5 and bRo5 compounds, respectively. The solid line equals the median and the dashed lines the quartiles. The dotted line marks 80% recovery.

Figure 8 presents the recoveries of compounds validly characterized via absorptive (A-to-B) (Figure 8, upper row) and secretory (B-to-A) (Figure 8, lower row) permeability in all three assay setups (*N* = 27; 67% bRo5). The compound set was the same as in 3.2, excluding invalid results (see definition in Figure 3) from either of the three setups. The violin plot analyses indicate a shift towards higher recoveries when BSA and pre-incubation (equilibrated method) are introduced, especially in the B-to-A direction. This finding is supported by the highest median recovery observed with the equilibrated method (Table 4). Notably, the equilibrated method resulted in an increased fraction of high recovery (>80%) for bRo5 compounds (72% vs. 50% for A-to-B and 94% vs. 39% for B-to-A, respectively, Table 5). The difference between A-to-B and B-to-A recovery was similar across methods (6.5–11%).

### 3.4. Comparative Analysis of P_app_ and Efflux Ratio of Reference Compounds with Known Human f_a_

**Figure 9:**
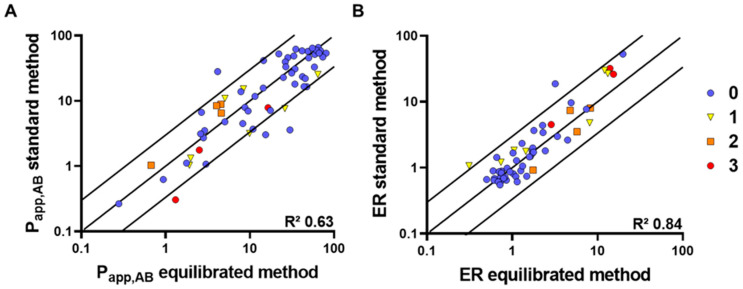
Comparison of P_app,AB_ (**A**) and ER (**B**) values obtained using the standard and the equilibrated method. The lines represent the line of unity ±3 fold. Compounds are classified based on their number of Lipinski rule violations. R^2^ of P_app,AB_ is 0.63 and R^2^ of ER is 0.84.

**Figure 10:**
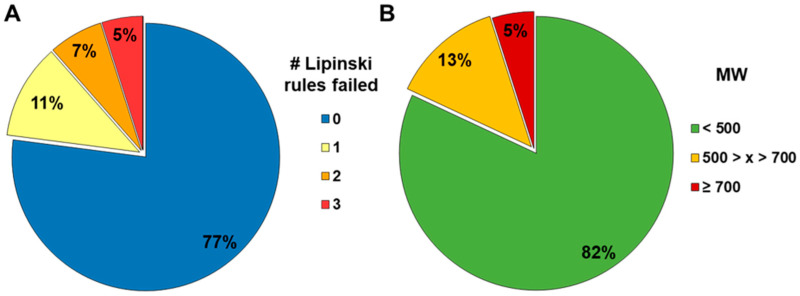
Composition of the comparative compound set with human f_a_ according to the number of failed Lipinski rules (**A**) and according to the binned molecular weight (**B**).

The standard method and the equilibrated method were used to assess the permeability of reference compounds with known human f_a_ (*N* = 61; Table 3). The P_app_ and ER of these compounds were compared directly between the two methods and plotted in Figure 9. The correlation coefficient (R^2^) for P_app_ and ER was 0.63 and 0.84, respectively. Additionally, differences of P_app_ and ER were mostly within three-fold magnitude of variation (92% and 97%, respectively). Differences between Ro5 and bRo5 were not apparent, likely due to the limited number of bRo5 compounds included in this set (Figure 10).

**Figure 11:**
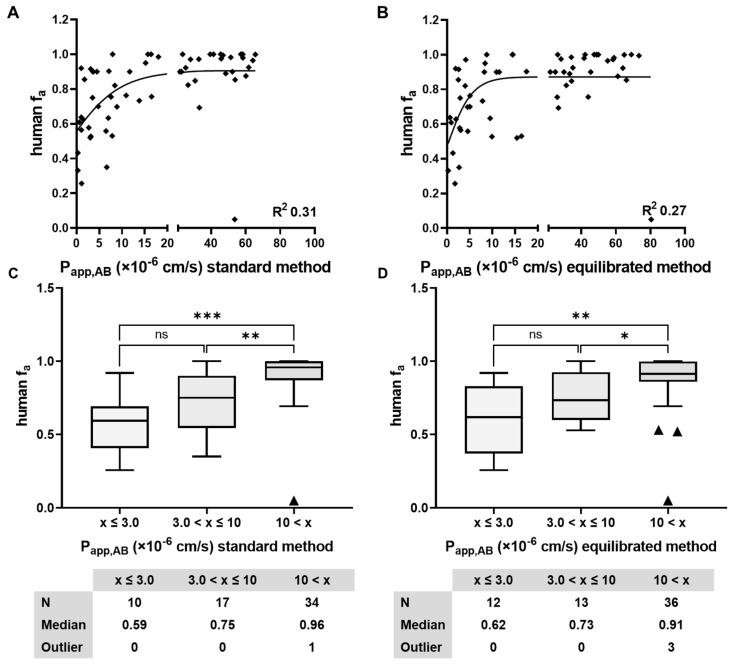
Sigmoidal fit and box plot analyses of binned P_app,AB_ values obtained from the standard (**A**,**C**) and the equilibrated method (**B**,**D**) for reference compounds with known human f_a_ (Table 3). R^2^ of P_app,AB_ of the standard method to human f_a_ is 0.31; R^2^ of the equilibrated method is 0.27. No significant difference was observed between the standard and the equilibrated method in all respective binning categories (low, medium, and high permeability). Diamonds represent single data points in the sigmoidal fit and triangles mark outliers of the respective binning category in the box plot analyses. Significance was calculated with Welch’s *t*-test, with significance defined at *p* < 0.05 (*), *p* < 0.01 (**), and *p* < 0.005 (***), ns refers to not significant.

A similar correlation of permeability bins (low, medium, and high) with the median f_a_ were observed (Figure 11), when comparing the P_app_ of the standard and equilibrated method to human f_a_. Compounds with low permeability, corresponding to P_app_ values < 3 × 10^−6^ cm/s, also had the lowest fraction absorbed (median f_a_ = 0.6), while compounds with a medium permeability and a corresponding P_app_ between 3 and 10 × 10^−6^ cm/s had median f_a_ values of 0.73 to 0.75. Compounds with high permeability corresponding to a P_app_ > 10 × 10^−6^ cm/s had a median f_a_ > 0.9. This comparison demonstrated equal performance of classification for both assays. However, it is worth noting that the compounds with published human f_a_ data available were mostly well-absorbed drug-like Ro5 substances with molecular weights of mostly <500 and <2 Lipinski violations (Figure 10). Therefore, the data set is biased towards these “simple” types of compounds. Nonetheless, this exercise confirmed the comparable performance of both methods in the assessment of permeability, which is challenging in a more complex chemical space where the standard method is limited due to a high rate of failure (3.2).

### 3.5. Permeability and Efflux Assessment of Internal Compounds and Comparison with In Vivo fafg

**Figure 12:**
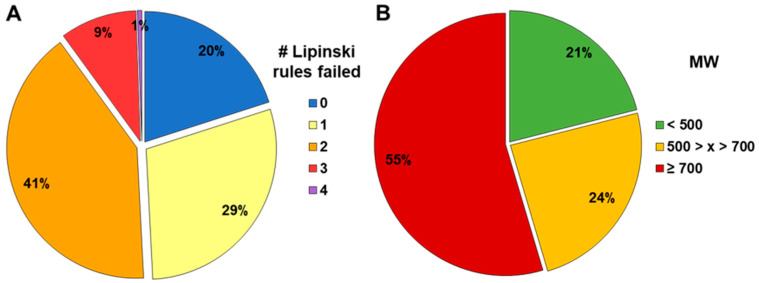
Composition of the internal compound set with rodent fafg according to the number of failed Lipinski rules (**A**) and according to binned molecular weight (**B**).

The equilibrated Caco-2 assay was used for permeability and efflux assessment of compounds with available internal rodent fafg data (*N* = 741). Compared to the compound set with published f_a_, the chemical space was different, more complex, and shifted towards the bRo5 space. Approximately 51% of the compounds violated two or more Lipinski rules, 79% possessed a molecular weight greater than 500 g/mol, and 55% even exceeded 700 g/mol (Figure 12).

**Figure 13:**
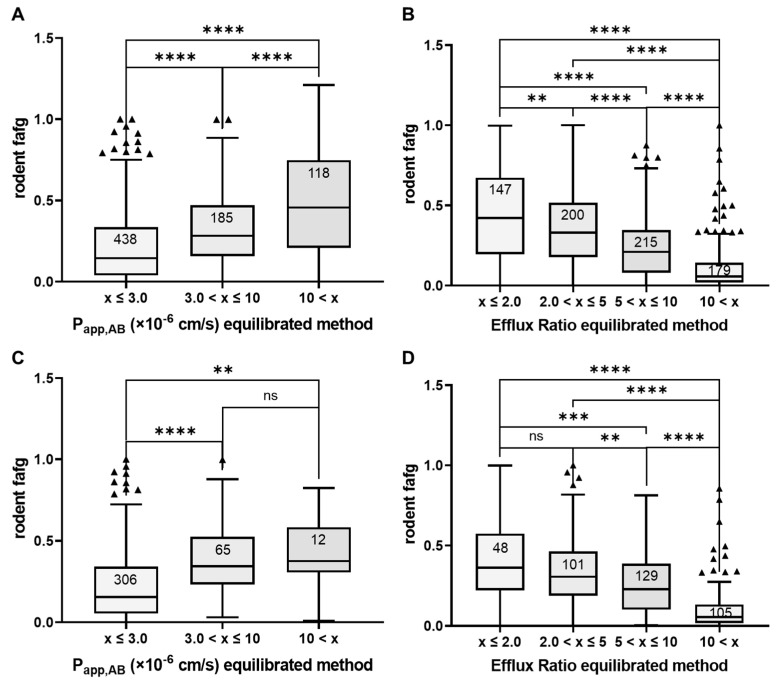
Boxplot analyses of binned P_app,AB_ (**A**,**C**) and binned ER (**B**,**D**) values obtained from the equilibrated method for compounds with internally determined rodent fafg. (**A**,**B**) consider Ro5 and bRo5 compounds (*N* = 741), whereas (**C**,**D**) only cover bRo5 compounds (*N* = 383). Triangles mark outliers of the respective binning categories. Significance was calculated with Welch’s *t*-test, with significance defined at *p* < 0.01 (**), *p* < 0.005 (***), and *p* < 0.001 (****), ns refers to not significant.

Applying the same cut-off permeability bins of low (P_app_ < 3 × 10^−6^ cm/s), medium (P_app_ > 3 and <10 × 10^−6^ cm/s), and high (P_app_ > 10 × 10^−6^ cm/s) values significantly differentiated the compounds into absorption (fafg) ranges (Figure 13A). Compounds with low permeability had a median fafg of 0.14, with medium permeability they demonstrated a median fafg of 0.3, and with high permeability the median fafg was 0.5. When filtered to bRo5 compounds (Figure 13C) in this data set, the outcome was comparable, but with the difference that the median fafg of the high P_app_ fraction (>10 × 10^−6^ cm/s) was 0.4, and thus slightly lower. However, given the smaller number of bRo5 compounds in this fraction (*N* = 12), this observation was not statistically significant.

**Figure 14:**
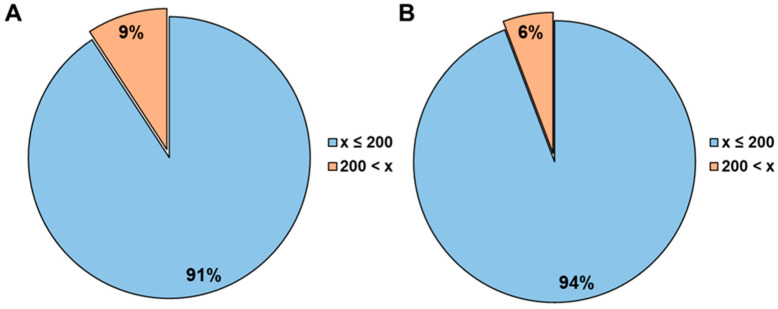
Binned solubility [µM] in phosphate buffer at pH 7.4 for a subset of compounds with corresponding internal solubility data available (*N* = 314 in total (**A**) and *N* = 174 bRo5 only (**B**)).

A big advantage of cellular permeability over non-cellular systems is that active transport mechanisms, such as efflux transport, can be covered. Because most bRo5 compounds possess efflux liability and are poorly soluble, efflux can have a significant impact on intestinal absorption. Indeed, in our investigated compound set with available solubility data (*N* = 317), most compounds (91% total and 94% of the bRo5s) are only soluble in phosphate buffer at less than 200 µM (Figure 14). At this low solubility and the predicted low concentration of dissolved drug in the gut after oral dosing, the intestinal efflux might not be completely saturated. Thus, in this case, efflux may limit oral absorption. Comparing the ER generated with the equilibrated method with rodent fafg, a correlation between increasing efflux and decreasing fafg is observed (Figure 13B). Although a reduction in fafg was already seen at ER > 2, the reduction became more significant at ER > 5. This observation was the same for the bRo5 fraction in this dataset (Figure 13D).

### 3.6. Categorization Strategies for Compound Ranking Using Equilibrated Caco-2 Permeability

Given the results from 3.5, it is possible to rank-order compounds early on, for example, prior to in vivo pharmacokinetic studies, and to define a fit-for-purpose in vitro setup as a selective assay. Although this is a retrospective study, the scenarios provide insight into potential prioritization strategies based on equilibrated Caco-2 permeability and efflux. Depending on the approach and the compound types of interest, fafg values higher than 0.5 might be mandatory for compound selection within a discovery project funnel. For other projects, for example with highly potent compounds, a target for fafg > 0.3 might be sufficient.

**Figure 15:**
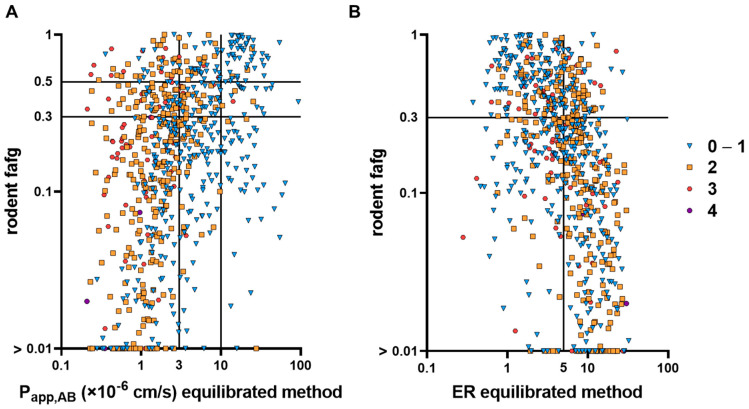
Compound ranking based on fafg and P_app,AB_ (**A**) or ER (**B**) cut-offs. Compounds are classified based on their number of Lipinski rule violations. *N* = 727 (total) and 369 (bRo5). If fafg was <0.01, values were set to 0.01. (**A**): P_app,AB_; true positive (upper-right-hand quadrant); true negative (lower-left-hand quadrant); false positive (lower-right-hand quadrant); and false negative (upper-left-hand quadrant). (**B**): Efflux ratios: true positive (upper-left-hand quadrant); true negative (lower-right-hand quadrant); false positive (lower-left-hand quadrant); and false negative (upper-right-hand quadrant).

Table 6 and Figure 15A show categorization approaches to identify compounds with the corresponding percentage of true and wrong predictions. Depending on the cut-offs, the number of true predictions ranged between 64% and 86% and was equal or slightly better for bRo5 compounds. The best predictability (86%) was reached when using a high permeability (P_app_ of 10 × 10^−6^ cm/s) and a high absorption (fafg 0.5) as cut-offs for bRo5 compounds. However, this represents a rather conservative approach and excludes many candidates that still had potentially reasonable fafg (only 1% true positive, compared to 5% when applying a P_app_ of 3 × 10^−6^ cm/s as cut-off). When considering that an fafg level down to 0.3 may still be acceptable, the true positive rates of 22% or 11% for all compounds and bRo5, respectively, are reached.

Applying an fafg cut-off of 0.5 drastically reduces the false negative rate, underlining the necessity of an individual evaluation of categorization. When using ER of 5 as a cut-off (Figure 15B, Table 7), true predictions of 68% were achieved with comparable distributions compared to the classification via P_app_.

## 4. Discussion

Over the past decades, the chemical space in pharmaceutical development of small molecules has shifted towards larger and more complex chemical entities, often with suboptimal physicochemical properties. This trend is caused by the intention to engage formerly non-druggable targets or to induce target degradation with bispecific molecules (PROTACs). Modern compounds often violate more than one of the Lipinski rules and are therefore referred to as beyond rule of five (bRo5) molecules [4]. Although the fundamental connection between permeability and absorption remains, it has become more relevant to characterize or rank permeability for compounds with lower absorption. As for efficiency reasons, throughout the pharmaceutical industry in vitro permeability assays have been developed for high-throughput and fast turnaround time, the standard incubation time is often too short to reach compound equilibrium in the cell, which limits the possibility of accurately measuring permeability and efflux. Cui et al. approached this challenge by introducing a 24 h pre-incubation, allowing the measurement of the intrinsic permeability at steady-state conditions. This pre-incubated setup achieved an adequate characterization of efflux and permeability for bRo5 substances, previously insufficiently or not characterized [11].

Besides this approach, several powerful tools based on physicochemical compound parameters have been established [4,5,6,8]. However, our goal was to develop a cell-based high-throughput method which provides the determination of active cellular permeability and efflux for compounds in the bRo5 chemical space, with a focus on the possibility to predict the properties or rank-order compounds according to their expected intestinal absorption based on this cellular assay. After the identification of the most appropriate assay parameters (Figure S1), a Caco-2-based system (parameters described in Section 2.5) was further optimized to cope with known challenges, including assay validity, recovery, and correlation to human f_a_ and rodent fafg.

To achieve a high-throughput and a rapid turn-around, we applied assay-ready cell culturing and maintained the cells for 7–8 days, in contrast to a more commonly described 21-day cultivation. The 7- and 21-day culturing revealed no significant difference in functional performance (Figure S2) and has already been shown to be suitable for advanced applications, like permeation studies with complex biorelevant media [19]. Furthermore, we intended to reduce the pre-incubation time from 24 h to 1 h for improved handling and to avoid the potential cytotoxic effects of test compounds. This was achieved by conducting the pre-incubation step without, and the main incubation step with, BSA to provide a higher fraction unbound for a faster equilibration of the system. The permeability and the ER in this method were comparable to a 24 h pre-incubation (Section 3.1; Figure 2) [11].

We compared the standard method (no pre-incubation, no BSA), the BSA-modified standard method (no pre-incubation, with BSA), and the equilibrated method (pre-incubation without BSA and main incubation with BSA; the exact method description is given in Section 2.5, Table 1) for assay validity and compound recovery with a compound set containing mainly bRo5 compounds. The introduction of BSA helped to reduce the number of invalid and qualified results with a valid result rate of 79% compared to 38% with the standard method. However, the equilibrated method exceeded the other two methods by achieving 94% valid results (Figure 6).

For shared valid results, there was a significant improvement in recovery using the equilibrated method, with 72% and 94% of the results with compound recovery of >80% for A-to-B and B-to-A permeability, respectively. In contrast, recoveries for both the standard method and the BSA-modified standard method were >80% for only 50% and 39% for A-to-B and B-to-A permeability, respectively.

Although the use of BSA is known to prevent non-specific binding and to improve compound recovery [10], the pre-incubation step of the equilibrated method further ameliorated the recovery through improved equilibration during the pre-incubation step and, consequently, steady-state compound transport. Adequate recovery rates (>80%) not only enhance the valid result rate but also boost confidence in cellular transport studies by ensuring the assay’s accuracy in measuring compound translocation across the layer.

The standard method and the equilibrated method were thoroughly compared regarding their output for permeability and efflux as well as their correlation to human f_a_. In a set of 61 compounds with known human f_a_ (Table 3) [17], 92% and 97% of predictions with P_app,AB_ and ER were within three-fold, respectively. P_app,AB_ to f_a_ between both methods was comparable, and compounds could be classified with no significant differences between the methods. It is important to highlight that the compounds with available human f_a_ data in the literature were predominantly well-absorbed Ro5 substances with molecular weights mostly below 500 and less than two Lipinski violations (Figure 10). The use of BSA in the main incubation of the equilibrated method did not have any significant impact on the permeability measurement. In other cell-based experimental setups, it was shown that the correlation dropped (R^2^ < 0.5) only at very low fractions unbound to plasma (f_u,p_ < 0.1%) (Figure S3). Despite the fact that the free drug hypothesis suggests stronger effects at lower protein binding, our evidence indicates that plasma proteins, like albumin, facilitate cellular uptake of compounds. This discrepancy arises from the dynamic and reversible nature of protein–compound interactions, as opposed to the static theoretical models [20]. Consequently, this dynamic interaction explains why the inclusion of BSA in cellular transport assays generally does not affect the outcome of the assay significantly [21].

Due to limited published data on human absorption of bRo5 substances from the existing literature, we applied the equilibrated method to 741 internal compounds with rodent fafg results. fafg served as a surrogate for in vivo absorption. In this compound set, 51% were bRo5 substances. In this analysis, a significant correlation between P_app_ and absorption was confirmed (Figure 13). Interestingly, the median absorption (fafg) of the compounds was lower compared to the published compounds with human f_a_ values when using the same binning. Because of the differences of the chemical entities, the larger AbbVie set contained a higher number of compounds with lower solubility (91%, Figure 14). Furthermore, many of these compounds were efflux substrates with ER greater than 2 (80%, Figure 13). The great number of compounds with efflux liability additionally drove a strong correlation between ER and absorption. Overall, P_app_ and ER both could be used to rank order compounds when applying individual cut-offs with a high rate of true absorption prediction (64–86%). Based on these results and considering that efflux is an active transport component (often missing in non-cellular models), the cellular equilibrated Caco-2 assay is a powerful supplement to close the gaps around the determination of P_app_ and ER for bRo5 compounds with suboptimal physicochemical properties. With this supplement we are now able to measure 94% of the current AbbVie chemical space reliably, while previously we could only determine P_app_ and ER of 38% of the studied compounds (Figure 6).

However, there are also limitations to this test system, as there is a significant fraction of false positives (predicted high fafg but low measured fafg) and false negatives (low permeability but high fafg) (Figure 15). As this cellular assay only takes permeability into account, the relevance of solubility and intestinal metabolism (f_g_) for oral absorption is missing. A compound with poor solubility or high intestinal metabolism may result in false positive results in our assay. On the other hand, higher absorption—despite low permeability—can be the result of either enabling formulation technologies in the corresponding in vivo studies or of particularities of the cellular system. Although binding effects to BSA are considered to be negligible in our assay systems (Figure S3), especially for compounds with a very low fraction that is unbound, high binding affinity to BSA, or even non-reversible covalent protein binding, may contribute to false positive results.

Taken together, the equilibrated Caco-2 assay provides a powerful cell-based in vitro tool for the characterization of difficult compounds within the bRo5 space, where simpler cellular systems or non-cellular in vitro assays have failed. Due to the complex nature of absorption, it is important to further consider properties like solubility and intestinal metabolism to improve the predictability of in vitro models for oral absorption. Employing the equilibrated Caco-2 assay in conjunction with in vitro assays evaluating solubility and intestinal metabolism in the context of a PBPK model allows for the best prediction of the outcome of human absorption in phase I clinical trials and beyond.

## 5. Conclusions

To tackle the challenges provided by complex chemical drug substances with suboptimal physicochemical properties, we developed an equilibrated Caco-2 method that reliably determines active cellular permeability and efflux for bRo5 compounds, with a specific focus on predicting and ranking compounds on their intestinal absorption. Through careful refinement of assay parameters, we optimized the Caco-2 system to overcome issues related to assay validity and compound recovery. By implementing an adapted 1 h pre-incubation step, we were able to achieve a faster equilibration process that demonstrated similar performance to a 24 h pre-incubation.

Overall, the equilibrated Caco-2 assay offers a powerful in vitro tool for characterizing challenging bRo5 compounds. To enhance the predictability and compound ranking for fafg, additional factors such as solubility and intestinal clearance need to be considered. Further research and consideration of these properties will improve the effectiveness of this assay in pharmaceutical development.

## Figures and Tables

**Table 1:** Details of standard method, BSA-modified standard method and equilibrated method in comparison.

	Standard Method	BSA-Modified Standard Method	Equilibrated Method
Compound concentration [µM]	1	3	3
Cassetting of compounds	Max. 3	No	No
Use of protein (BSA)	No	Yes	Yes (Main incubation only)
Pre-incubation	No	No	Yes (60 min)
Main incubation	60 min	60 min	60 min
Compound tune prior to assay	No	No	Yes

**Table 2:** Table of compounds for the investigation of the pre-incubation setup. bRo5: 2 or more Lipinski rules violated.

Name	MW	cLogP	Lipinski Classification
Amiloride	230	−0.5	Ro5
Cimetidine	252	−0.1	Ro5
Carbamazepine	236	2.8	Ro5
Metoprolol	267	1.8	Ro5
Atenolol	266	0.4	Ro5
Quinidine	324	2.5	Ro5
Propranolol	259	2.6	Ro5
Verapamil	455	5	Ro5
Midazolam	326	3.3	Ro5
Prazosin	383	1.7	Ro5
Compound 1	>700	>3	bRo5
Compound 2	<700	>3	Ro5
Compound 3	>700	>3	bRo5
Compound 4	>700	n/a	bRo5
Compound 5	<700	>3	bRo5
Compound 6	>700	>3	bRo5
Compound 7	>700	<3	bRo5
Compound 8	>700	<3	bRo5
Compound 9	>700	>3	bRo5
Compound 10	>700	>3	bRo5
Compound 11	>700	n/a	bRo5

**Table 4:** Median percent compound recovery and mean differences between A-to-B and B-to-A transport of compounds in different assays. The first values are the fraction of all compounds, the second of Ro5, and the third of bRo5 compounds.

Assay	Recovery A-to-B Median			Recovery B-to-A Median			Mean Difference A-to-B and B-to-A
	Total	Ro5	bRo5	Total	Ro5	bRo5	
Standard method	98	100	79	84	93	75	11
BSA-modified standard method	89	94	79	83	92	78	6.5
Equilibrated method	91	101	86	94	97	92	8.2

**Table 5:** Percent compound recovery of compounds in different assays. The fraction of compounds with percent recovery >80% in the respective setup is shown. The first values are the fraction of all compounds, the second of Ro5 and the third of bRo5 compounds.

Assay	Recovery A-to-B [>80%]			Recovery B-to-A [>80%]		
	Total	Ro5	bRo5	Total	Ro5	bRo5
Standard method	67	100	50	59	100	39
BSA-modified standard method	67	100	50	59	100	39
Equilibrated method	81	100	72	93	100	94

**Table 6:** Percent of true and false predictions using P_app_ and fafg cut-offs.

	All Compounds			bRo5 Only		
P_app_/fafg cut-off	3/0.3	3/0.5	10/0.5	3/0.3	3/0.5	10/0.5
True positive (%)	22	13	7	11	5	1
True negative (%)	42	53	72	58	71	85
False positive (%)	20	28	9	11	16	1
False negative (%)	16	6	12	20	8	13
Total true (%)	64	66	79	69	76	86

**Table 7:** Percent of true and false predictions using ER and fafg cut-offs.

	All Compounds	bRo5 Only
ER/fafg cut-off	5/0.3	5/0.3
True positive (%)	29	20
True negative (%)	39	48
False positive (%)	20	17
False negative (%)	12	15
Total true (%)	68	68

## Data Availability

The raw/processed data required to reproduce these findings cannot be shared at this time due to legal or ethical reasons.

## Supplementary Materials

The following supporting information can be downloaded at: https://www.mdpi.com/article/10.3390/pharmaceutics16070846/s1, Figure S1: Overview of assay modifications. Figure S2: Comparison of P_app,AB_ and ER generated with 7-day or 21-day Caco-2 cells. Figure S3: Influence of f_u,p_ on P_app,AB_. References [12,22] are cited in the Supplementary Materials.
