# Supplementary material for: Conquering the beyond Rule of Five Space with an Optimized High-Throughput Caco-2 Assay to Close Gaps in Absorption Prediction
Source: Pharmaceutics. 2024 Jun 22;16(7):846. doi: 10.3390/pharmaceutics16070846 (PMC11280027; doi:10.3390/pharmaceutics16070846)
Supplement: Supplementary file 1 [file pharmaceutics-16-00846-s001.zip › pharmaceutics-3033067-supplementary.pdf]

Supplementary Figures

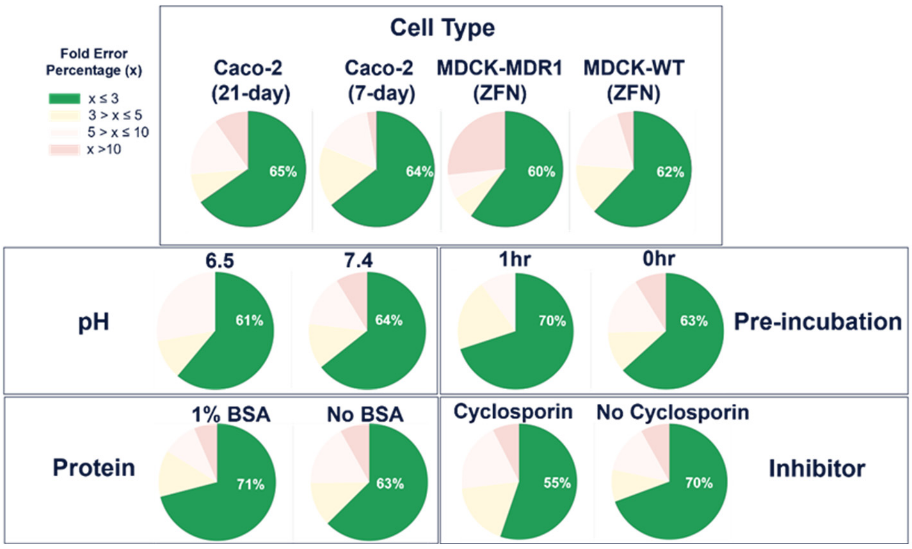

Figure S1. Overview of assay modifications. Assay modifications were tested through pairwise analysis of shared compounds to determine the most suitable basic method assay parameters. The variables that were investigated were the following: the in vitro system (Caco-2, MDCK_MDR1_ZFN [22], and MDCK_WT_ZFN [22]), pH, pre-incubation, addition of protein, addition of inhibitor. The corresponding assay endpoints were log-transformed and normalized to the respective human f_a data using linear correlation. For comparison, fold deviation to human f_a was used, and superior conditions were highlighted.

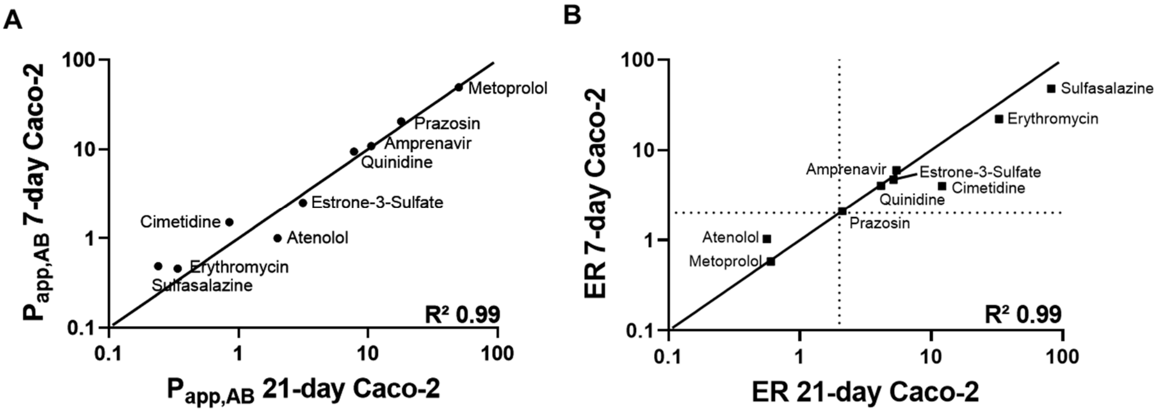

Figure S2. Comparison of $P_{app,AB}$ and ER generated with 7-day or 21-day Caco-2 cells. When using Caco-2 cells for transport studies, the traditional approach over the last decades was to maintain the cells for 21 days after seeding into transwell plates. This cultivation period ensures full recovery and differentiation of the monolayer. However, nowadays, specialized assay-ready cells that are pre conditioned via gentle freezing and thawing techniques exhibit a better fitness and recover faster, which not only allows for assay-ready seeding but also reduces cultivation times on the transwell plates [12]. For this manuscript, we used assay-ready Caco-2 cells for all assays conducted. To confirm their fitness and performance after shortened incubation times, bidirectional transport studies were carried out using nine well-known reference compounds (metoprolol, atenolol, prazosin, quinidine, amprenavir, cimetidine, estrone-3-sulfate (E3S), erythromycin, and sulfasalazine). The cells were seeded into transwell plates and either maintained for 21 days or for only

7 days before being used in an assay. The comparisons of apparent permeability (P_{app}) in the apical to basolateral direction **(A)** and obtained efflux ratios (ER) **(B)** of the nine reference compounds are displayed in Figure S2. P_{app} and ER values were highly comparable between 7-day and 21-day Caco-2 cells, evidenced by an R^2= 0.99 in both cases. All compounds known to be efflux transporter substrates were correctly identified as such with both cell culture methods, as they exhibited ER > 2 (prazosin, quinidine, amprenavir, cimetidine, E3S, erythromycin, and sulfasalazine). Since the shortened cultivation time of 7 days revealed the same performance as the 21-day cultivation time for the Caco-2 cells, all further experiments were conducted using cells maintained for 7–8 days.

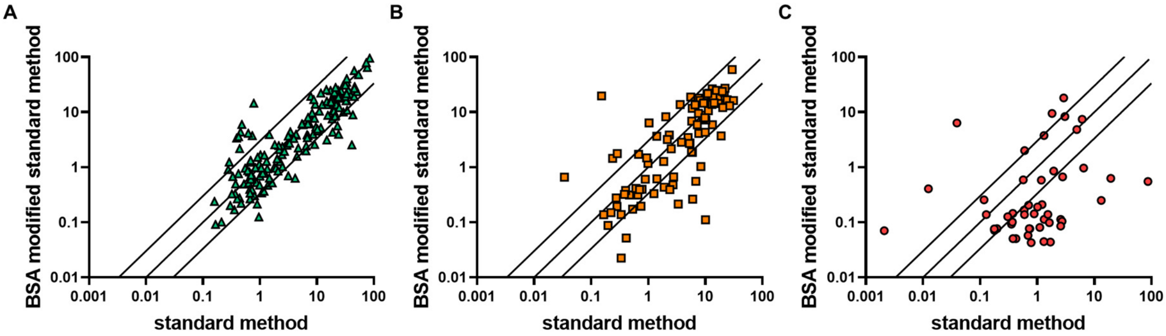

Figure S3. Influence of $f_{u,p}$ on $P_{app,AB}$. A comparison was made between permeability results obtained using the standard method and the BSA-modified standard method (Table 1), with dependence on the fraction unbound in plasma ($f_{u,p}$), using MDCK_MDR1_ZFN cells as described in [22], instead of Caco-2 cells (*N* = 370). (**A**) shows $P_{app,AB}$ correlation between standard and BSA-modified standard method when $f_{u,p}$ > 0.01, (**B**) when $f_{u,p}$ 0.001 < x ≤ 0.01, and (**C**) when $f_{u,p}$ ≤ 0.001. A good correlation between the methods was observed, with R^2 > 0.5 and > 70 % of compounds being within 3-fold variation for those with $f_{u,p}$ > 0.001 (0.1%). However, for compounds with lower $f_{u,p}$, the correlation significantly decreased.
